# Deciphering myeloid-derived suppressor cells: isolation and markers in humans, mice and non-human primates

**DOI:** 10.1007/s00262-019-02302-2

**Published:** 2019-01-25

**Authors:** Luca Cassetta, Espen S. Baekkevold, Sven Brandau, Anna Bujko, Marco A. Cassatella, Anca Dorhoi, Carsten Krieg, Ang Lin, Karin Loré, Olivia Marini, Jeffrey W. Pollard, Mikael Roussel, Patrizia Scapini, Viktor Umansky, Gosse J. Adema

**Affiliations:** 1 MRC Centre for Reproductive Health, Queen’s Medical Research Institute, The University of Edinburgh, 47 Little France Crescent, EH16 4TJ Edinburgh, UK; 2 Centre for Immune Regulation, Department of Pathology, University of Oslo, Oslo University Hospital, Rikshospitalet, Oslo, Norway; 3 West German Cancer Center, University Hospital Essen, University of Duisburg-Essen, Essen, Germany; 4 Division of General Pathology, Department of Medicine, University of Verona, Verona, Italy; 5 Friedrich-Loeffler-Institut, Federal Research Institute for Animal Health, Greifswald, Insel Riems Germany; 6 Faculty of Mathematics and Natural Sciences, University of Greifswald, Greifswald, Germany; 7 Department of Immunology, Max Planck Institute for Infection Biology, Berlin, Germany; 8 Department of Microbiology and Immunology, Hollings Cancer Center, Medical University of South Carolina, Charleston, USA; 9 Department of Medicine Solna, Immunology and Allergy Unit, Karolinska Institutet, Stockholm, Sweden; 10 Center for Molecular Medicine, Karolinska Institutet, Stockholm, Sweden; 11 Centre Hospitalier Universitaire, Pôle Biologie, INSERM, UMR U1236, Université Rennes 1, EFS Bretagne, Rennes, France; 12 Skin Cancer Unit, German Cancer Research Center (DKFZ), Heidelberg, Germany; 13 Department of Dermatology, Venereology and Allergology, University Medical Center Mannheim, Ruprecht-Karl University of Heidelberg, Mannheim, Germany; 14 Radiotherapy and OncoImmunology Laboratory, Department of Radiation Oncology, Radboud Institute for Molecular Life Sciences, Radboud University Medical Center, Geert Grooteplein 28, 6500 HB Nijmegen, The Netherlands

**Keywords:** Human, Mouse, Non-human primates, Myeloid-derived suppressor cells, Mye-EUNITER

## Abstract

In cancer, infection and inflammation, the immune system’s function can be dysregulated. Instead of fighting disease, immune cells may increase pathology and suppress host-protective immune responses. Myeloid cells show high plasticity and adapt to changing conditions and pathological challenges. Despite their relevance in disease pathophysiology, the identity, heterogeneity and biology of myeloid cells is still poorly understood. We will focus on phenotypical and functional markers of one of the key myeloid regulatory subtypes, the myeloid derived suppressor cells (MDSC), in humans, mice and non-human primates. Technical issues regarding the isolation of the cells from tissues and blood, timing and sample handling of MDSC will be detailed. Localization of MDSC in a tissue context is of crucial importance and immunohistochemistry approaches for this purpose are discussed. A minimal antibody panel for MDSC research is provided as part of the Mye-EUNITER COST action. Strategies for the identification of additional markers applying state of the art technologies such as mass cytometry will be highlighted. Such marker sets can be used to study MDSC phenotypes across tissues, diseases as well as species and will be crucial to accelerate MDSC research in health and disease.

## Introduction

Pathological events such as cancer, infection and inflammation profoundly alter the homeostasis of organisms and activate robust immune responses. Usually these events are also accompanied by increased myelopoiesis that results in an emergency supply of myeloid cells [1, 2]. These myeloid cells are innate immune cells that provide one of the first lines of defense against pathogens or neoplastic insults and play a fundamental role in immune surveillance, antigen presentation and T-cell activation.

However, conditions like chronic inflammation, autoimmune disease and cancer cause the aberrant expansion of myeloid cells that are phenotypically and functionally distinct from normal myeloid cells and facilitate rather than halt disease progression [3]. In the context of the Mye-EUNITER COST action (http://www.mye-euniter.eu) we refer to these cells as myeloid regulatory cells (MRC) as the heterogeneous group of myeloid cells that have acquired immunoregulatory and/or immunosuppressive activity, usually as a consequence of the disease of the host. The term “regulatory” is used with reference to the far better characterized regulatory T cells, which in contrast to classical T cells are not immune effector cells, but rather downregulate immune responses [4]. Examples of regulatory myeloid cells include, but are not limited to immunosuppressive granulocytes, tolerogenic dendritic cells (DC), macrophages and myeloid-derived suppressor cells (MDSC), further subdivided in monocytic MDSC (M-MDSC) and polymorphonuclear MDSC (PMN-MDSC).

The MDSC represent a heterogeneous population of myeloid cells that fail to complete their regular differentiation to macrophages, granulocytes or DC under physiological conditions like aging [5] or pathological conditions like chronic inflammation or cancer, although we can not exclude that they are in part derived from their mature counterparts [6–8]. They are derived from bone marrow hematopoietic precursors due to the altering of myelopoiesis by sustained production of inflammatory mediators [9–11].

The characterization of the subtypes of pathologically expanded myeloid cells in different diseases, model systems and species has generated a considerable amount of data regarding markers for their isolation and methods to study their function for the different organisms as well as on how to distinguish pathological subsets such as MDSC from immune protective myeloid cells. Unfortunately, due to the differences in marker and model selection, the information available in the literature, even within one species, is highly heterogeneous and frequently conflicting observations are reported. Therefore, proper standardization of MDSC identification isolation and functional characterization are essential to guide the field [12].

In this review we will summarize the efforts of the network in the form of a panel of markers for the identification of MDSC in mouse, human and non-human primates models; moreover we will discuss critical aspects of the isolation and study of MDSC that need to be standardized to avoid artifacts and allow meaningful data comparison across laboratories.

## Isolation and characterization of major MDSC types from peripheral blood

### Human monocytes and M-MDSC

Human monocytes in peripheral blood can be isolated either through elutriation, magnetic beads separation or gradient centrifugation. Monocytes are readily identifiable among the HLA-DR^+^ CD11b^+^ myeloid compartment, as they constitute 10–20% of all peripheral blood mononuclear cells (PBMC) obtained from standard density gradient centrifugation. Blood monocytes, however, still consist of a phenotypically and functionally heterogeneous populations of cells that are conventionally divided into 3 subsets based on the expression of CD14 and CD16 [13]. The major CD14^high^CD16^neg^ classical monocyte population is rapidly recruited to sites of inflammation or tissue damage, while the less-frequent CD14^low^CD16^high^ non-classical monocytes exhibit vascular surveillance functions during steady state. The CD14^high^CD16^dim^ “intermediate” monocytes [14] are the least abundant monocyte population, although their abundance can vary in pathological conditions [15]. Recent studies of gene expression profiles of the monocyte subsets [16–18] and of their kinetics in blood [19] have shown that the monocyte population in blood is a developmental continuum. A minor fraction of classical monocytes differentiates in blood into intermediate monocytes that further transition into non-classical monocytes [19]. In case a clear segregation of non-classical and intermediate monocytes is key, it is advised to use proper isotype controls in FACS sorting. Moreover, since natural killer (NK) cells may express CD16, it is crucial to include an NK-marker like CD56 (or CD335/NKp46) together with lymphoid lineage markers (CD3 and CD19) in the “dump channel”. Of note, a recent study compared the frequency and phenotypes of monocytes extracted from whole blood and gradient stratification, and found that the relative frequency of classical (CD14^high^CD16^neg^) versus non-classical (CD14^low^CD16^high^) monocytes was significantly different [13]. Thus it is important to disclose the source of blood monocytes for useful comparisons of data.

Human M-MDSC are present in the same density fraction as monocytes but differ from monocytes by low or the absence of HLA-DR expression. They are further characterized as lymphocyte lineage marker negative cells with the following phenotype CD11b^+^HLA-DR^−^CD14^+^CD15^−^. It is possible to use CD33 myeloid cell marker instead of CD11b. In this case, M-MDSC display high CD33 expression relative to PMN-MDSC [20]. M-MDSC induction and expansion are mediated by a combination of soluble factors (e.g., VEGF, GM-CSF, M-CSF, S100A8/A9, IL-4, IL-6, IL-10, PGE2, MMP9, CXCL5, CXCL12 and C5a) produced by tumor and/or surrounding cells such as stromal cells, T cells or macrophages [21]. These factors essentially trigger activation of members of the STAT family of proteins, such as STAT3, STAT6 and STAT1, ultimately leading to expression of genes involved in the blockade of myeloid differentiation and in genes with immune regulatory activity.

Expansion of immunosuppressive M-MDSC populations is observed in different cancer types including breast, colorectal cancer, melanoma, glioma and more, indicating that tumor derived factors can systemically activate this population in the blood of cancer patients [22–25]. For a detailed critical review on MDSC in cancer we refer the reader to the companion review by Umansky et al. [26] in this symposium-in-writing series.

### Human neutrophils and PMN-MDSC

Human peripheral blood neutrophils from healthy donors typically sediment on top of erythrocytes after density gradient centrifugation (see Table 1 for human M-MDSC and PMN-MDSC isolation in blood). While centrifuging peripheral blood of patients with acute and chronic inflammatory conditions (cancer, sepsis, infections, autoimmune diseases), many studies reported the presence of low-density neutrophils (LDN, as opposed to the normal density neutrophils, NDN) within the mononuclear cell fraction [27–29]. These LDN display typical granulocyte markers (e.g., CD66b and CD15) and show neutrophil-like morphology. They are composed of a mixture of immature neutrophils at different differentiation stages as well as of mature neutrophils with an activated phenotype [27–29]. A substantial part of these LDN consist of immunosuppressive PMN-MDSC. These PMN-MDSC have been found in patients with cancer [20, 28], HIV infection [30–32], trauma, [33], sepsis [34, 35], but also in individuals with an altered immune status, like pregnant women [36–39] or people receiving G-CSF for stem cell mobilization [40–42]. Human PMN-MDSC are typically described as CD66b^+^CD15^+^CD14^−/dim^CD33^dim^HLA-DR^−^ cells [20, 28, 43], a phenotype closely similar to NDN. Studies have described PMN-MDSC as being composed of immature neutrophils [44, 45], heterogeneous populations consisting of both immature and mature “neutrophil-like” populations [20, 35, 37, 46], or even “activated/degranulated” mature neutrophils [30, 32–34, 47–51].

**Table 1:** Guidelines for the isolation of human M-MDSC and PMN-MDSC in blood

Variable	Indication
Time	Isolate cells within one hour after blood withdrawal, avoid use of frozen samples
Anticoagulant	Collect peripheral blood in either ethylenediaminetetraacetic acid (EDTA) or sodium citrate containing tubes
Separation reagent	Use commercially available gradient solutions (1.077 g/L)

Besides the lineage markers CD66b or CD15, the inclusion of maturation markers such us CD16 and CD11b can be used to discriminate CD11b^high^CD16^high^ mature neutrophils from neutrophil precursors present within PMN-MDSC [44]. Alternatively, CD10 can be used as a marker to identify mature neutrophils within heterogeneous PMN-MDSC populations, instead of CD16 [41].

PMN-MDSC have been shown to also express other markers, including activation markers (e.g., CD62L, CD54/ICAM-1, CD63, CD274/PD-L1), chemokine receptors (e.g., CXCR2, CXCR4) and functional markers [e.g., arginase 1(ARG1) and Lectin-like oxidized low-density lipoprotein (LDL) receptor-1 (Lox-1)], at variable levels depending on the disease type and severity [20, 29].

**Fig. 1:**
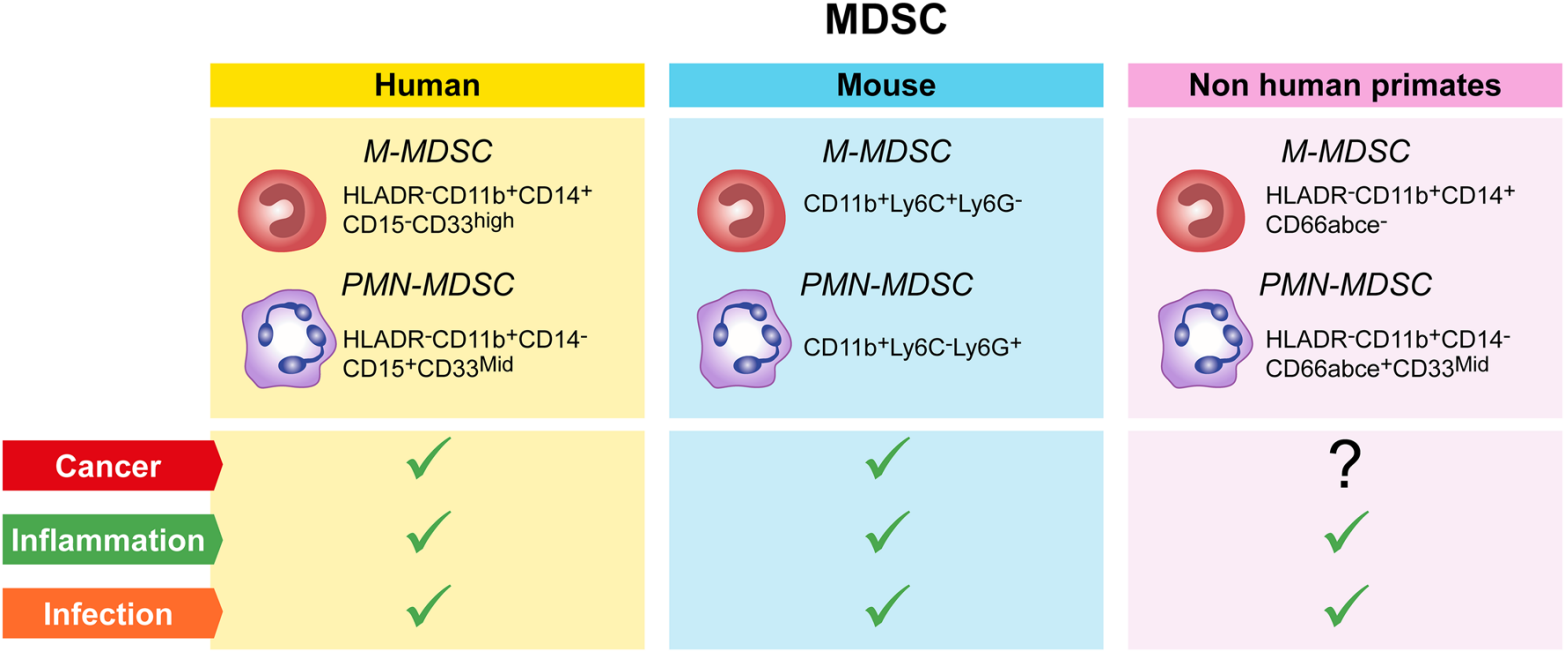
MDSC populations and their markers in humans, mice and non-human primates. Each colored box (yellow = human, blue = mouse, pink = non-human primates) contains the markers for the identification of M and PMN-MDSC, in blood after density centrifugation. These cells are mainly found in the blood of patients or animals with cancer, inflammatory diseases or infection (green ticks); so far no reports indicated a correlation between M and PMN-MDSC and cancer in NHP (question mark)

However, the precise origin of immunosuppressive PMN-MDSC, altered granulopoiesis and/or new functional properties acquired by mature neutrophils in response to disease-specific factors remains to be determined. Thus, defining specific immunophenotypic and functional markers that will allow researchers to unequivocally discriminate the features of circulating and tissue infiltrated immunosuppressive PMN-MDSC from their normal counterpart is of key importance. The importance of standardization experiments has been recognized by colleagues in the field and first data sets have been obtained [43, 52, 53]. In this same context, the COST consortium has recently agreed on a minimal number of immunophenotypic markers required as a first step to identify circulating PMN-MDSC in blood (Fig. 1). In addition, a number of published procedures to collect and manipulate blood for PMN-MDSC and M-MDSC recovery has been evaluated as part of the activities of the Mye-EUNITER COST consortium. The conclusions reached from this comparison have resulted in a number of experimental guidelines and a minimal marker panel summarized in Table 1 and Fig. 1. Currently, the consortium is in the process of further validating the selected markers across laboratories and performing experiments applying high-end technologies to answer key questions regarding population homogeneity and definition of additional functional markers.

### Murine M-MDSC and PMN-MDSC

Murine MDSC have initially been defined to express Gr1 and CD11b surface molecules. Further dichotomy is based on the differential expression of the cell surface molecule Ly6C and Ly6G as they define two major subsets: CD11b^+^Ly6G^−^Ly6C^hi^ M-MDSC that share phenotypical and morphological characteristics with monocytes, and CD11b^+^Ly6G^+^Ly6C^lo^ PMN-MDSC, which resemble neutrophils [6, 7, 43, 54]. These markers are present on multiple hematopoietic cells thereby calling for a careful exclusion gating for stringent flow cytometric identification of the MDSC subsets. A dump channel for identifiers unique to lymphocytes, dendritic cells, and distinct eosinophilic granulocytes (NK1.1, NKp46, CD3, CD19/220, CD11c, CCR3, Siglec-F) is advisable. Of note, recent identification of a subset of suppressive eosinophils (also coined as MDSC) further complicates the flow cytometric identification of PMN-MDSC [55]. Whereas in humans and non-human primates (NHP) MDSC are primarily characterized in the blood, in mice these cells are often investigated in a tissue context, besides a few exceptions [46]. This analysis adds additional layers of complexity. Tissue confounding factors, primarily tissue resident phagocytes, may be minimized by a rigorous multiparameter/multidimensional study design. For instance, for analysis of MDSC in lung tumors, besides addition of the hematopoietic CD45 to the marker panel as above, inclusion of the alveolar macrophage marker Siglec-F ensures distinction of M-MDSC from these non-MDSC cell types. Further expansion of the phenotype panel by inclusion of functional markers, primarily Arg1 and iNOS, also contributes as well to the characterization of these MDSC subsets. However, unique phenotypic markers have not been specifically assigned to murine MDSC implying that functional studies are critical for definition of this MDSC subset [12].

Differentiation of MDSC from other myeloid cells, including bonafide monocytes and neutrophils, tumor associated macrophages (TAM) and tumor associated neutrophils (TAN) is currently challenging. In diseases associated with expansion of the MDSC it appears impossible to make a phenotypic distinction between inflammatory monocytes and M-MDSC. Cell surface markers, as well as the buoyant properties of such cells are overlapping [56]. TAM exhibit certain markers, which may be employed to distinguish them from M-MDSC. TAM express F4/80 and lack or show low expression of Ly6C, abundant transcripts coding for IRF8, M-CSF and reduced ER-stress markers [53, 57, 58]. CD115 and CD244 are expressed by subsets of PMN-MDSC infiltrating tumors and are absent on *bonafide* neutrophils [59]. In addition, the buoyant properties of the murine PMN-MDSC appear distinct from neutrophils [46, 56]. Additional studies are nevertheless required to substantiate such phenotypic discrepancies between PMN-MDSC and neutrophils and validate their value in phenotypic studies.

The Mye-EUNITER network analyzed several published reports on MDSC phenotyping [43, 52, 60] and agreed on a minimal list of markers for the identification of M- and PMN-MDSC in mouse (Fig. 1); multiple mouse clones are used and no recommendations are given beyond the markers. Validation of the suppressive activity is essential given the challenges in immune phenotyping.

### Non-human primate M-MDSC and PMN-MDSC

NHP are unique animal models due to their close genetic and physiological similarities to humans. One of major NHP species used are rhesus macaques (*macaca mulatta*), which are critical in several late state preclinical investigations prior to testing in humans [61]. The immune cell subsets are largely similar in phenotype, function, and distribution between rhesus macaques and humans. Phenotyping of rhesus immune cells by flow cytometric analysis is highly feasible due to overlap of surface molecules and cross-reactivity of most anti-human antibodies [62]. The NHP Reagent Resource website (http://www.nhpreagents.org) is a useful tool to search for reported cross-reactive human antibody clones. However, some myeloid cell subsets like MDSC have only just started to be defined in rhesus macaques.

To purify MDSC from rhesus blood, commercially available gradient solutions (1.077 g/L) are suitable to isolate rhesus PBMC by standard gradient centrifugation. M-MDSC accumulates in the interphase after centrifugation. As in human blood, the population of neutrophils that also appears within the interphase is referred to as LDN [46]. Like in humans, NDN sediment to the bottom together with erythrocytes after the separation procedure and can be purified using a 3% dextran sedimentation assay [63]. This procedure works well for both human and rhesus samples.

Recent studies by one of the Mye-EUNITER consortium members tested an array of antibodies to identify rhesus MDSC, along with confirmation for cross-reactivity of different clones (Table 2) [64]. Due to differences in the phenotypes of myeloid cell subsets from humans and rhesus macaques plus the lack of a few cross-reactive antibodies, the marker sets that are used to identify human MDSC do not work properly to define the rhesus counterparts. Differences in the phenotype of immune cells between human and rhesus macaques include CD56, a unique lineage-specific antigen of human NK cells that is also present on a subset of monocytes in rhesus macaques. As CD8 is expressed on rhesus NK cells, however, it can be used together with CD3 and CD20 to exclude lymphoid cells in the gating strategy to identify rhesus MDSC. Likewise, NKG2A or NKp46 are suitable markers of NK cells in rhesus macaques. CD14 works well for identification of monocytes and M-MDSC in both rhesus and humans [64, 65]. Although CD66abce and CD15 identify the same neutrophil population in humans [65], the anti-CD66abce antibody (clone: TET2) is most commonly used to stain rhesus neutrophils. CD33 is one of the key markers used to identify human MDSC as it is highly expressed on human M-MDSC and intermediately expressed on PMN-MDSC. However, most commercial anti-CD33 antibodies are not cross-reactive with rhesus macaques, and the only clone able to recognize CD33 on rhesus cells only stains rhesus granulocytes and not monocytes or myeloid dendritic cells [64]. Gene expression analysis of purified rhesus cells is essential to determine CD33 mRNA expression in these cells. Currently, the best way to discriminate M-MDSC and monocytes is, therefore, based on the absence of expression of HLA-DR on the M-MDSC. For detection of rhesus PMN-MDSC, CD33 can be used as a marker. Of note, in the total rhesus LDN population both CD33^−^ and CD33^+^ cells are present, of which only the CD33^+^ cells show inhibition of T cell responses and thus represent the PMN-MDSC [64]. Like in humans, the rhesus CD33^−^ LDN is still a heterogeneous population composed of immature neutrophil precursors. The phenotypic and functional heterogeneity within the LDN population in rhesus macaques suggest that also here the definition of PMN-MDSC has yet to be optimally defined and is in need for additional functional markers.

**Table 2:** Minimal marker panel for the identification of non-human primates’ M-MDSC and PMN-MDSC in blood

Surface marker	Antibody clone	Additional cross-reactive clones
HLA-DR	Tü36	L243, G46-6
CD3	SP34-2	SK7, FN18
CD20	L27	2H7
CD8	RPA-T8	SK1
CD33	AC104.3E3	
CD11b	ICRF44	
CD66abce	TET2	
CD14	M5E2	MoP9

## MDSC identity requires functional assays and surrogate markers

Myeloid cells can be called MDSC only if they show T cells suppressive functions; for a detailed critical review on MDSC functional assays and suppressive pathways active in M-MDSC and PMN-MDSC we refer the reader to the companion review by Bruger et al. in this symposium-in-writing series [12].

As an additive strategy to functional MDSC analysis, flow cytometry analysis of functional markers on the cells directly ex-vivo, so without in vitro manipulation, may provide insight into their functional potential. As reviewed by Bruger et al. [12], MDSC suppression is mediated by various distinct mechanisms but so far the immunosuppressive molecules involved are largely overlapping between mouse and human MDSC while being less well defined in non-human primates. Analysis of the following suppressive molecules/pathways may turn out to be rewarding: (i) upregulation of Arg1 expression, leading to the deprivation of arginine, which is critical for the proper expression of the TCR zeta-chain and coupling of TCR-mediated antigen recognition to diverse signal transduction pathways [6, 7, 11]; (ii) production of nitric oxide (NO) via activation of inducible NO synthase (iNOS) causing the nitration of T-cell receptors (TCR) and chemokines important for T-cell migration or induction of T-cell apoptosis [66, 67]; (iii) synthesis of reactive oxygen species (ROS) [6, 9]; (iv) production of IL-10 and transforming growth factor (TGF-β) inhibiting immune effector cell functions [7, 68]; (v) upregulation of programmed death-ligand 1 (PD-L1) [69], which inhibits T cell-mediated reactivity via interaction with PD-1 receptor expressed on T cells [70]; (vi) upregulation of ectonucleotidases CD39 and CD73 [71] resulting in increased production of adenosine that suppresses effector T cell functions [72]; (vii) increased expression of Fas ligand, mediating T-cell apoptosis [73]; (viii) expression of elevated levels of indoleamine 2,3-dioxygenase (IDO) that degrade L-tryptophan, causing cell cycle arrest and anergy in T cells or skewing T-cell differentiation towards regulatory T cells (T_reg_) [74–76].

Finally, LOX-1 has been proposed as a candidate marker to distinguish human immunosuppressive PMN-MDSC from normal neutrophils in blood and tissues from cancer patients [53, 77] and in blood from infants [78]. These studies demonstrated that only the LOX-1^+^, but not LOX-1^−^, neutrophils displayed the characteristic gene signature and immune suppressive activity typical of PMN-MDSC [53, 77, 78]. Although very intriguing, these observations need to be further validated in independent patient cohorts. However, as much as surrogate markers simplify analysis of challenging samples they should ideally be performed in parallel with standardized functional assays. If impossible they should preferentially be controlled using marker expression on other myeloid cells in the same donor in the same assay.

In summary, identification of specific markers or combinations that are able to unequivocally define MDSC populations with an immunosuppressive phenotype in blood and tissues remains one of the major challenges in the MDSC field.

## Challenges for the characterization and localization of MDSC in tissues

The introduction of therapies targeting the immune system to fight diseases, like immune checkpoint inhibitors to treat cancer, shows that a deep understanding of the immune cell composition in human blood and disease tissue is essential for guiding the development of immunotherapy. Moreover, knowledge of the immune cells that encompass and invade tumors could predict the success or failure of therapy. However, establishing robust disaggregation protocols that are reproducible among laboratories is challenging, since different tissue samples require variably aggressive treatments, which have to be established empirically. However, some aspects of tissue dissociation are amenable to standardization, most notably those related to enzyme types, blends and activity. Most protocols depend on the use of collagenase, which is available in many different formats exhibiting highly variable substrate activities. Some products, however, offer standardized blends of purified collagenases with little lot variation and reduced levels of endotoxins [79]. Application of such collagenase blends is a prerequisite for attempts towards consistent flow cytometric assessment of tissue cells, most notably by enhancing reproducible release of cellular subsets and allowing confident analysis of cellular markers, as variability of enzymatic epitope cleavage (or generation of neo-epitopes) is strongly reduced. However, these issues should still be considered with the introduction of new lots, with parallel digestion treatment of control cells prior to antibody staining. Furthermore, the addition of DNAse is critical, as dying cells will release DNA that may trap viable cells, and greatly reduce cellular yields. It should be pointed out that neutrophils/TAN are more sensitive to enzymatic exposures and isolation procedures than other myeloid subsets. Immunosuppressive “neutrophil-like cells” have been identified in human tissues, such as in the spleen [80–82], in the placenta [83] and in tumor tissue (the latter neutrophil population are generally defined as “tumor infiltrating/associated neutrophils, TAN) [53, 84]. As previously suggested by Quatromoni et al. [85], the application of optimized disaggregation method and enzymatic cocktails may, therefore, be necessary to maximally preserve the vitality, effector functions and cell-surface marker expression of neutrophil/TAN population recovered from tissues. Finally, application of density gradient fractionation to increase the frequency of myeloid cells following digestion should be performed with caution. The density and buoyancy of myeloid tissue cells may be significantly different to their blood counterparts, for which most gradient centrifugation protocols are developed, and artifacts related to cell recovery are easily introduced. Instead, combinations of antibodies to lineage-restricted or stroma-associated antigens in a “dump-mix” for negative gating could be considered.

Moreover many studies have shown that not only the composition of immune cell subsets in tissues, but also their spatial distribution, is crucial for their function. Such information is lost when samples are analyzed by cytometric techniques, and systematic assessments of MDSC in situ are needed to fully understand their biology. Indeed, pioneering work by Galon and colleagues showed the impact and predictive value of immune cell localization inside or at the tumor margins for the prognosis of cancer patients [86]. However, standardized histological characterization of cells is challenging due to the highly diverse tissue processing protocols employed by different research labs, and the comparatively low number of parameters that may be simultaneously analyzed (4–5 markers). Yet, several of the marker combinations proposed for flow cytometric analysis may be employed on cryopreserved or formalin-fixed specimens, following proper antigen retrieval treatment. In addition, emerging methods and technologies for highly multiplexed immunohistochemical analysis, like consecutive immunostaining and destaining of single histological slides [87] or application of spectral unmixing of fluorescent signal emission to accommodate separation of a large array of fluorophores on a single specimen [88], combined with biologically interpretable machine learning algorithms that enable unbiased image analysis [87] will tremendously increase the number of parameters for analysis.

## Conclusions and future perspectives

Myeloid cells show high plasticity and readily adapt to changing conditions such as those present in cancer, infection and inflammation. As a consequence many myeloid cells can acquire (immune) regulatory activity. A prototypic example among those “myeloid regulatory cells” are MDSC, which are characterized by their profound immunosuppressive activity and key pathophysiological importance. One of the aims of the Mye-EUNITER COST consortium is to standardize markers and protocols to study these different MDSC to provide the scientific community with better tools to analyze these cells, distinguish functional subsets and ultimately decipher their important role in health and disease. Recent data using high-end approaches such as mass cytometry have confirmed the crucial role of MDSC and other myeloid cells with regulatory activity in disease and their potential as targets for therapy as well as biomarkers for therapy response prediction. Antibody based imaging of key functional molecules (e.g., Arg1, NOX2, iNOS, PD-L1, IL-10, TGF-β, CD124) and the post-translational protein modifications like the phosphorylation of transcription factors (e.g., phospho-STATs and cEBPβ) as present in myeloid cells will further complement these data sets. Such high-resolution imaging data can then be used for 3D reconstruction of intact tissue [89] and for studying cell–cell interactions, phenotypes, and microenvironments [90]. These approaches are expected to be particularly valuable to uncover the complexity of myeloid cell biology, especially when combined with (single-cell) transcriptomic- and epigenetic analysis. Ultimately, these investments should yield a comprehensive atlas of the complex functional relationships between multiple immune-cell subsets in a pathological tissue context [43].

Understanding immune regulation at this integrative level will be key in the development of personalized approaches for immunotherapy of cancer.

## Abbreviations

Arg1: Arginase-1
DC: Dendritic cell
EU: European Union
iNOS: Inducible nitric oxide synthase
LDN: Low density neutrophil(s)
LOX-1: Lectin-like oxidized low-density lipoprotein (LDL) receptor-1
MDSC: Myeloid-derived suppressor cell(s)
M-MDSC: Monocytic myeloid-derived suppressor cell(s)
MRC: Myeloid regulatory cell(s)
NDN: Normal density neutrophil(s)
NHP: Non-human primate(s)
PMN-MDSC: Polymorphonuclear myeloid-derived suppressor cell(s)
ROS: Reactive oxygen species
SCF: Stem cell factor
TAM: Tumor associated macrophage(s)
TAN: Tumor associated neutrophil(s)
